# Gender-Specific Differences of Dental Emergency Patients and the Use of Antibiotics: A 4-Year Retrospective Study

**DOI:** 10.3290/j.ohpd.a44037

**Published:** 2020-07-04

**Authors:** Jens Weusmann, Helena Schmitt, Benedikt Braun, Kawe Sagheb, Brita Willershausen, Benjamin Mahmoodi

**Affiliations:** a Dentist, Department of Operative Dentistry of the University Hospital of the Johannes Gutenberg-University, Mainz, Germany. Designed and coordinated the study; statistical analysis and writing the paper; read and approved the final manuscript.; b Dentist, Department of Operative Dentistry of the University Hospital of the Johannes Gutenberg-University, Mainz, Germany. Carried out the study; read and approved the final manuscript.; c Dentist, Department of Operative Dentistry of the University Hospital of the Johannes Gutenberg-University, Mainz, Germany. Carried out the study; read and approved the final manuscript.; d Dentist, Department of Prosthodontics of the University Hospital of the Johannes Gutenberg-University, Mainz, Germany. Statistical analysis and writing the paper; read and approved the final manuscript.; e Dentist, Department of Operative Dentistry of the University Hospital of the Johannes Gutenberg-University, Mainz, Germany. Coordination of the study; read and approved the final manuscript.; f Dentist, Department of Operative Dentistry of the University Hospital of the Johannes Gutenberg-University, Mainz, Germany. Designed and carried out the study; statistical analysis and writing the paper; read and approved the final manuscript.

**Keywords:** antibiotics, dental emergencies, emergency patients, gender dentistry

## Abstract

**Purpose:**

To evaluate the clinical characteristics of dental emergency patients with special regard on gender-specific differences related to the utilisation and use of antibiotics.

**Materials and Methods:**

A retrospective analysis of all patients who presented to the emergency service of a university hospital in from 2010 to 2013 was performed. Demographic data, diagnosis, conducted treatment and the prescription of antibiotics were recorded and further analysed.

**Results:**

Altogether, 16,296 patients visited the emergency service. Of these patients, only one-fourth (25.7%; n = 4185) suffered from a diagnosis with urgent treatment needs. Gender-specific differences were found in the reason of visit. Males presented significantly more often with severe diagnoses, like abscess or trauma. Females presented significantly more often with non-urgent diagnoses, not directly connected to oral hygiene habits, like temporomandibular disorders (TMD), denture sore or dolor post extractionem. Moreover, an overuse of antibiotics was found among emergency patients, with every fifth patient (20.2%; n = 3291) being prescribed an antibiotic.

**Conclusion:**

Better public education on dental emergencies and constant updates for dentists about the use of antibiotics in dental emergency care is necessary to secure adequate medical supply for severe dental emergencies and to avoid an inappropriate use of antibiotics.

Dental emergencies are defined as all forms of maxillofacial and dental traumas, secondary bleedings after dental surgeries and odontogenic infections^11^ which often imply the urgency of an immediate treatment to prevent further complications such as tooth loss^5,12,29^ or the spread of odontogenic infections, which can result in life-threatening complications.^2,4,24,25^ Next to these severe emergencies, the German Society for Dental and Oral Medicine (Deutsche Gesellschaft für Zahn-, Mund- und Kieferheilkunde – DGZMK)^11^ differentiates relative, non-acute, mostly pain-related dental emergencies without severe consequences.

In Germany, the dental emergency service is available outside the dentists’ regular consulting hours including nights, weekends and public holidays. It is performed by private practices and is regulated by each state in Germany and by the university dental clinics. In the Rhine-Main area with 5.5 million inhabitants, these would be the universities of Frankfurt and Mainz.

Former studies have shown that emergency visits, especially at night-time have increased.^1,21^ Furthermore, the number of traumatic dental injuries have increased within recent years, with the highest incidence in the late evenings and at weekends,^16,19^ due to factors associated with the modern lifestyle.^10,14,18^

Mostly, patients seek care due to pain, caused by infections that are confined within the tooth, which can often be managed by local operative treatment. Although there is evidence that antibiotics do not release tooth pain^8^ and they are indicated for the adjunct treatment of overt odontogenic infections,^3^ an inappropriate use of antibiotics among dentists with an increase of prescriptions has been reported,^9,17,22^ especially for emergency treatment.^7^

Most surveys on dental out-of-hours services only include a small number of cases or are often limited to certain selected diagnoses, mostly dental trauma^1,27^ and odontogenic infections.^6^ Other possible causes of tooth pain, such as pulpitis, caries decay, periodontal disease or loss of restorations, are not considered.

Previous studies found that men are more likely to visit emergency centres.^1,15,21^ The gender difference is even more pronounced in severe infections which required hospitalisation.^13^ However, there are no data available regarding the entire spectrum of dental emergency visits and gender-specific differences in relation to the corresponding diagnoses.

The purpose of this study was to examine the clinical characteristics of all dental emergency visits to the dental emergency service of the University hospital of the Johannes-Gutenberg University in Mainz, Germany, and to give an overview of the reason of utilisation, with special regard to gender differences, focusing not only on patients with urgent emergencies. Furthermore, the implemented therapies, especially the use of antibiotics, were evaluated.

## MATERIALS AND METHODS

All patients that presented to the dental emergency service of the University Medical Center in Mainz, Germany from January 2010 to December 2013 were included in the study. The opening hours were from Monday to Thursday, 17.00–24.00, Friday 15.00–24.00 and Saturday, Sunday and legal holidays 8.00–24.00. About 60–75 active dentists work in the University Medical Center Mainz; out of these, 30–40 routinely participate in the dental emergency service. The service is open both for regular patients of the University Medical Center and for patients from external facilities. A retrospective investigation of the patients was carried out, analysing demographic data (age, gender, day and month of visit) as well as the diagnosis made by the dentist on duty, the conducted treatment and use of antibiotics. Multiple diagnoses were possible. Information was extracted from electronic clinical database (SAP, Walldorf, Germany/Visident, Wolfsburg, Germany) and subjected to further analysis.

According to the hospital law (Landeskrankenhausgesetz, §§36,37) of the state of Rhineland-Palatinate, Germany, no ethical approval is necessary in retrospectively performed studies evaluating already existing patient data. All patients were informed about the anonymised use of their records at the time they underwent treatment in the hospital.

Patients in need of hospitalisation in the Department of Oral and Maxillofacial Surgery were not considered.

Collected data were recorded in Microsoft Excel 2010 (Redmond, WA, USA). Descriptive statistics were computed using SPSS 22 (IBM, Armonk, NY, USA); chi-square test was used for analysing distribution between groups.

## RESULTS

### Demographic Data

Altogether, 16,296 patients with dental problems visited the emergency service department between January 2010 and December 2013.

**Fig 1:**
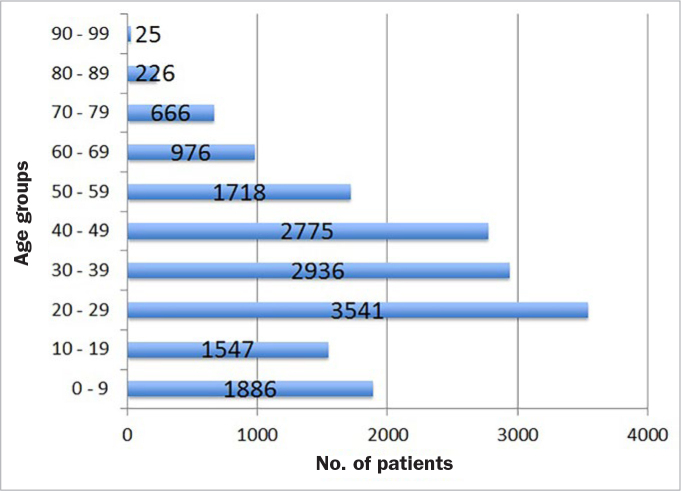
Distribution of patients by age group.

Of them, 8856 patients (54.3%) were male and 7440 (45.7%) female, with a male to female ratio of 1.2:1. The average age was 35.5 ± 19.5 years (standard deviation), ranging from 1 month to 98 years. The age distribution peak was between 20 to 29 years (n = 3451; 21.7%) followed by 30 to 39 years (n = 2936; 18.0%). Altogether, more than 57% of the patients were between 20 and 49 years old (Fig 1).

**Fig 2:**
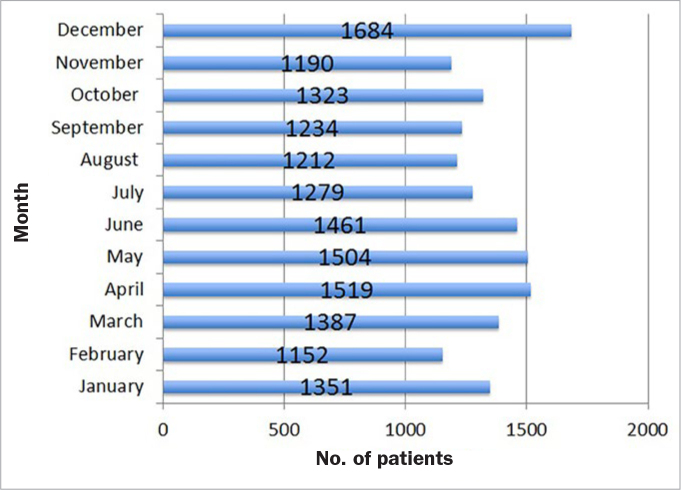
Distribution of patients by months of the year.

**Fig 3:**
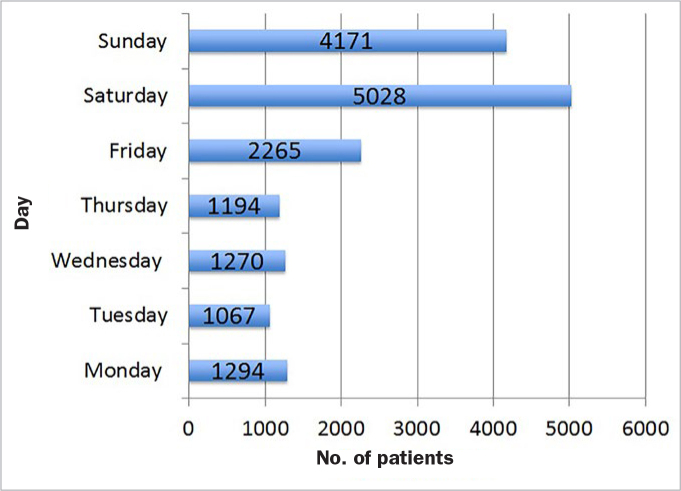
Distribution of patients by days of the week.

The distribution of visits among the months of year was homogeneous, with December being the most frequently visited month (n = 1684; 10.3%) and February with least visits (n = 1152; 7.1%, Fig 2). Saturday was the busiest day of the week, with nearly one-third of all visits (n = 5028; 30.1%), Tuesday the least busy with 1067 visits (6.5%). More than half of all patients (n = 9199; 56.6%) visited on weekends (Fig 3). Altogether, 1075 patients (6.6%) sought dental care on a national holiday.

The mean number of visits per day was 7 among the days of the week and 22 at weekends.

Nearly half of all trauma patients (47.1%; n = 615) presented on a weekend.

### Diagnoses

Main reason for emergency visits was apical periodontitis (n = 3928; 24.2%), followed by abscess (n = 1911; 11.7%) and postendodontic pain (n = 1590; 9.8%). Only one-fourth of the patients (n = 4185; 25.7%) came due to a diagnosis with urgent treatment needs: 11.7% (n = 1911) with abscess, 8% with trauma (n = 1305), 3.0% with a pericoronitis and 2.9% with a postoperative bleeding (n = 477). Three out of four patients presented to the emergency service without an urgent treatment need.

While young patients suffered more often from trauma, pericoronitis and orthodontic complications, older patients came more often with oral bleedings, the loss or damage of definitive or temporary restorations as well as impaired wound healing and implant complications.

A severe diagnosis such as apical periodontitis, abscess, sinusitis, dental trauma and soft-tissue injury was significantly more often seen among men, while females were significantly more often affected by temporomandibular disorders (TMD), dolor post-ex, denture sore, mucosal abnormalities, impaired wound healing and orthodontic complications.

The mean age varies between the diagnoses (Table 1).

**Table 1:** Diagnosis in relation to demographic characteristics

Diagnosis	Patients	Mean age (years)	Gender		
			Male n (%)	Female n (%)	P value
TMJ contusion	37	27.1 ± 20.7	21 (56.8)	16 (43.2)	0.411
Maxillary sinusitis	51	34.9 ± 14.1	17 (33.3)	34 (66.7)	0.017*
Sialadenitis	61	40.2 ± 18.1	40 (65.5)	21 (34.4)	0.051
Malocclusion	62	36.9 ± 15.5	25 (40.3)	37 (59.7)	0.128
Denture sore	65	63.5 ± 15.2	25 (38.5)	40 (61.5)	0.007*
Implant complication	94	54.5 ± 16.2	41 (43.6)	53 (56.4)	0.023
Impaired wound healing	114	41.6 ± 18.2	45 (39.5)	69 (60.5)	0.001*
Mucosa abnormalities	179	35.5 ± 21.7	79 (44.1)	100 (55.9)	0.004*
Orthodontic complication	199	18.1 ± 10.4	81 (40.7)	118 (59.3)	< 0.001*
Loss/damage filling	263	34.7 ± 13.9	145 (55.1)	118 (44.9)	0.422
Loss/damage of temporary restoration	321	47.2 ± 17.3	174 (54.2)	147 (45.8)	0.502
Follow-up treatment	328	40.1 ± 18.9	171 (52.1)	157 (47.9)	0.440
TMD	428	36.5 ± 17.8	167 (39.0)	261 (61.0)	< 0.001*
Loss/damage restoration	454	50.9 ± 14.5	222 (48.9)	232 (51.1)	0.010*
Oral bleeding	477	53.6 ± 22.4	276 (57.9)	201 (42.1)	0.064
Pericoronitis	492	26.3 ± 9.1	240 (48.8)	252 (51.2)	0.007*
Dolor post-ex	593	36.9 ± 16.9	241 (40.6)	352 (59.4)	< 0.001*
Soft-tissue injury	938	19.8 ± 21.7	591 (63.0)	347 (37.0)	< 0.001*
Pulpitis	1102	33.2 ± 15.0	634 (57.5)	468 (42.5)	0.015*
Trauma	1305	14.7 ± 15.7	784 (60.1)	521 (39.9)	< 0.001*
Periodontal disease	1326	44.3 ± 17.3	735 (55.4)	591 (44.6)	0.212
Postendodontic pain	1590	36.2 ± 15.5	754 (47.4)	836 (52.6)	0.040*
Abscess	1911	38.2 ± 19.0	1119 (58.6)	792 (41.1)	< 0.001*
Apical periodontitis	3928	38.0 ± 16.1	2258 (57.5)	1670 (42.5)	< 0.001*
Other/Unknown	517	36.8 ± 17.4	256 (49.5)	261 (50.5)	0.826

### Treatment and the Use of Antibiotics and Analgesics

Out of 16,296 patients who visited in the dental emergency service, 3291 (20.2%) received a prescription for antibiotics. The most frequently prescribed antibiotic was amoxicillin (n = 1170; 53.7%) followed by a combination of amoxicillin and clavulanic acid (n = 850; 25.8%) and clindamycin (n = 246; 7.5%).

6.8% (n = 1108) of patients were treated with antibiotics only, or with a combination of antibiotics and analgesics without local treatment; 2022 patients (12.4%) received a combination of antibiotics and local treatment. Most patients (n = 11122; 68.3%) were treated locally without further antibiotic treatment. Among patients with absolute emergencies, more than one-third of the patients received an antibiotic (n = 1561; 37.7%) and 19.3% (n = 2381) of the patients with ‘relative’ emergencies.

The prescription of antibiotics was highest among patients with abscess (59.7% of cases), sialadenitis (52.2%) and pericoronitis (40.7%), while its use for apical periodontitis (29.9%), periodontal diseases (21.0%) and pulpitis (8.2%) was more restrictive. The 3.6% (n = 585) patients treated with antibiotics only were predominantly diagnosed with apical periodontitis (34.7%), abscess (29.7%) or postendodontic pain (5.1%).

Analgesics were prescribed in 3313 cases (20.3%) with ibuprofen being the most frequently prescribed drug (n = 2377; 71.7%). In 6.1% of cases, analgesics were the only therapy, while a combination of analgesics and antibiotics as the only treatment was conducted in 523 patients (2.3%; see Table 2).

**Table 2:** Use of antibiotics and treatment by diagnosis

Diagnosis	Patients	Antibiotics n (%)	Analgesics n (%)	Local treatment n (%)
Jaw fracture	37	12 (32.4)	6 (16.2)	1 (2.7)
Maxillary sinusitis	51	9 (17.6)	14 (27.5)	8 (15.6)
Sialadenitis	61	32 (52.2)	6 (9.8)	4 (6.5)
Malocclusion	62	0 (0)	5 (8.1)	48 (77.4)
Denture sore	65	4 (6.2)	2 (3.1)	59 (90.8)
Implant complication	94	17 (18.1)	8 (8.5)	65 (69.1)
Impaired wound healing	114	35 (30.7)	26 (22.8)	64 (56.1)
Mucosa abnormalities	179	11 (6.1)	16 (8.9)	98 (54.7)
Orthodontic complication	199	2 (1.0)	3 (1.5)	188 (94.5)
Loss/damage filling	263	3 (1.1)	28 (10.6)	221 (84.0)
Loss/damage of temporary restoration	321	4 (1.2)	2 (0.6)	306 (95.3)
Follow-up treatment	328	30 (9.1)	18 (5.5)	253 (77.1)
TMD	428	12 (2.8)	139 (32.5)	20 (4.6)
Loss/damage restoration	454	5 (1.1)	6 (1.3)	392 (86.3)
Oral bleeding	477	23 (4.8)	19 (4.0)	451 (94.5)
Pericoronitis	492	200 (40.7)	143 (29.1)	439 (89.2)
Dolor post-ex	593	194 (32.7)	134 (22.6)	398 (67.1)
Soft-tissue injury	938	212 (22.6)	116 (12.4)	620 (66.1)
Pulpitis	1102	90 (8.2)	222 (20.1)	843 (76.5)
Trauma	1305	200 (15.3)	156 (12.0)	748 (57.3)
Periodontal disease	1326	279 (21.0)	304 (22.9)	1130 (85.2)
Postendodontic pain	1590	318 (20.0)	354 (22.3)	1290 (81.1)
Abscess	1911	1140 (59.7)	592 (31.0)	1517 (79.4)
Apical periodontitis	3928	1175 (29.9)	1138 (29.0)	2802 (71.3)
Other/Unknown	517	43 (8.3)	164 (31.7)	284 (54.9)

## DISCUSSION

Our data give an overview over a large patient collective in an industrial country. All patients who visited the dental emergency service of a university hospital centre in a metropolitan were included to work out demographical and clinical characteristics of patients seeking for care.

In contrast to other studies, our data show a rather balanced distribution of emergency visits between male and female patients. Previous studies found that men are more likely to visit emergency centres.^1,15,21^ Most of these studies investigate a certain diagnosis (like infections or trauma) and include only a particular group of patients, while in our study, all patients who presented to the emergency service were included. Regarding specific diagnoses, we found that men are significantly more often affected by trauma, abscesses, apical periodontitis and pulpitis which is in agreement with the literature. This difference may be explained by the fact that males are more likely to neglect oral hygiene^26^ and show a lower use of preventive health services than females.^28^ Men rather wait for the consultation of a doctor, since the infections has run to an acute course.

Diagnoses which are not directly connected to oral hygiene habits are significantly higher among women, like orthodontic complications, postendodontic pain and pericoronitis as well as TMD, dolor post extractionem and mucosa abnormalities. Females seem to visit the emergency service even for non-urgent reasons due to safety reasons.

Regarding the age of patients, those suffering from dental trauma (14.7 ± 15.7 years) showed the lowest average age of all groups. This finding is consistent with the literature, showing that children are more prone to trauma and that dental trauma appears more frequently among children and adolescent.^1,10,19^ The oldest mean age was found among patients with prosthodontic problems, like denture pressure sores (63.5 ± 15.2 years) or implant complications (54.5 ± 16.2 years), followed by oral bleeding (53.6 ± 22.4 years), which is associated with age-related oral and general diseases.

Our results do not show a strong seasonal variation of emergency visits throughout the year, as it was found in former publications, which showed an increase during warm weather and summer holidays.^1,15^ We found a small peak between April and June, followed by a decrease of visits during the major holiday season. December is the month with the highest visit rates, which might result from the German healthcare system and the closure of many private practices at the end of the year.

Regarding the weekly variation, more than half of all patients (n = 9199; 56.6%) and nearly half of all trauma patients (47.1%; n = 615) came on Saturdays and Sundays. The number of patients per day was three times higher on weekends (22 patients) than on weekdays (7 patients). This high frequentation results from private practices closure and more recreational activities and sport on weekends.

Our data indicate that the majority of patients who visited the emergency service do not have an urgent treatment need (n = 12111; 74.3%) as they are defined by the DGZMK*, which stands in contrast to studies from other countries, where most patients suffered from a severe disease,^1^ while other studies from Germany show a similar share of non-urgent visits.^6^ This might be explained by the fact that basic and emergency treatments are fully covered by the insurance companies and that the demand in health is very high in Germany, so that the term ‘emergency’ is defined differently by patients in Germany. The high number of patients without urgent treatment needs make the organisation of emergency services more difficult.

The quantity can lead to waiting times for patients with severe diagnoses, to an impairment of prognosis and an increase of complications, especially among trauma patients who often need immediate intervention to save the injured teeth. Public education about dental emergencies, better prevention and structural adjustments of the service are necessary to avoid this rush on the emergency service to secure adequate medical supply to patients. The weekend-peak of visits could be compensated with a higher number of medical staff on these days and a better collaboration with the private practices’ emergency service.

Relating to guidelines, adjunctive systemic antibiotic treatment is indicated in acute abscesses in medically compromised patients, systemic involvement (fever, trismus), progressive infections and dislocation and soft-tissue injuries.^20^ The number of prescriptions of antibiotics (in 20.2% of cases) in this study was lower than reported in other studies, but should still be regarded as critical. Although other studies showed a higher use of antibiotics^7,23^ and less frequent local treatment, the number of prescriptions in our study still seems pretty high, regarding the rare indications. The university hospital in Mainz is a maximum care hospital and the only university hospital in the State of Rhineland-Palatine. Therefore, many medically compromised and high-risk patients are referred to the hospital from practical doctors and the private practices emergency service due to the high complication risk. This might explain a higher use of antibiotics in the emergency care of the university hospital, but cannot be the reason for every fifth patient to receive an antibiotic.

The literature shows an inappropriate use of antibiotics, especially in emergency centres. Tulip et al reported that 50% of patients were treated with antibiotics alone without any local treatment,^23^ while Daily et al reported even 62%.^7^ In our survey, 6.8% of patients received an antibiotic as the sole therapy. Education about an appropriate use of antibiotics and an update of dentists’ knowledge is mandatory to avoid unnecessary application of antibiotics.

All data were evaluated retrospectively from the electronic records of the patients and results depend on documentation, which was made by the dentist on duty. 3% of patients (n = 517) did not have a definitive diagnosis. Patients’ medical history was not considered in the study, which sure has an impact on the indication for antibiotic prescription. Furthermore, it is not reported whether patients who presented to the emergency service were already under antibiotic treatment.

## CONCLUSION

The majority (75%) of emergency patients have no urgent treatment need, which might result in disadvantages for real emergency patients. There seem to be gender-specific differences in the use of the dental emergency service regarding the diagnoses. Males are more often affected by severe diagnoses, possibly resulting from neglecting oral hygiene, while females more often present with diagnoses not directly connected to oral hygiene habits. Despite available guidelines, our data indicate an overuse of antibiotics among dental emergency patients. Patient education of the purpose of dental emergencies, as well as an update for dentists about the use of antibiotics is necessary for the organisation and optimisation of emergency services to secure adequate medical supply for patients in need.

## References

[ref1] Bae JH, Kim YK, Choi YH (2011). Clinical characteristics of dental emergencies and prevalence of dental trauma at a university hospital emergency center in Korea. Dent Traumatol.

[ref2] Baqain ZH, Newman L, Hyde N (2004). How serious are oral infections?. J Laryngol Otol.

[ref3] Brennan MT, Runyon MS, Batts JJ, Fox PC, Kent ML, Cox TL (2006). Odontogenic signs and symptoms as predictors of odontogenic infection: a clinical trial. J Am Dent Assoc.

[ref4] Bucak A, Ulu S, Kokulu S, Oz G, Solak O, Kahveci OK (2013). Facial paralysis and mediastinitis due to odontogenic infection and poor prognosis. J Craniofac Surg.

[ref5] Bucher K, Neumann C, Thiering E, Hickel R, Kuhnisch J (2013). Complications and survival rates of teeth after dental trauma over a 5-year period. Clin Oral Investig.

[ref6] Cachovan G, Phark JH, Schon G, Pohlenz P, Platzer U (2013). Odontogenic infections: an 8-year epidemiologic analysis in a dental emergency outpatient care unit. Acta Odontol Scand.

[ref7] Dailey YM, Martin MV (2001). Are antibiotics being used appropriately for emergency dental treatment?. Br Dent J.

[ref8] Fedorowicz Z, van, Zuuren EJ, Farman AG, Agnihotry A, Al-Langawi JH (2013). Antibiotic use for irreversible pulpitis. Cochrane Database Syst Rev.

[ref9] Germack M, Sedgley, CM, Sabbah W, Whitten B (2017). Antibiotic use in 2016 by members of the American Association of Endodontists: report of a national survey. J Endod.

[ref10] Glendor U (2009). Aetiology and risk factors related to traumatic dental injuries – a review of the literature. Dent Traumatol.

[ref11] Hausamen J (1995). Welche therapeutische Maßnahmen sind im zahnärztlichen Notdienst indiziert?. DGZMK.

[ref12] Hecova H, Tzigkounakis V, Merglova V, Netolicky J (2010). A retrospective study of 889 injured permanent teeth. Dent Traumatol.

[ref13] Hwang T, Antoun JS, Lee KH (2011). Features of odontogenic infections in hospitalised and non-hospitalised settings. Emerg Med J.

[ref14] Lam R, Abbott P, Lloyd C, Lloyd C, Kruger E, Tennant M (2008). Dental trauma in an Australian rural centre. Dent Traumatol.

[ref15] Lygidakis NA, Marinou D, Katsaris N (1998). Analysis of dental emergencies presenting to a community paediatric dentistry centre. Int J Paediatr Dent.

[ref16] Petersson EE, Andersson L, Sorensen S (1997). Traumatic oral vs non-oral injuries. Swed Dent J.

[ref17] Preus HR, Fredriksen KW, Vogsland AE, Sandvik L, Grytten JI (2017). Antibiotic-prescribing habits among Norwegian dentists: a survey over 25 years (1990–2015). Eur J Oral Sci.

[ref18] Sandalli N, Cildir S, Guler N (2005). Clinical investigation of traumatic injuries in Yeditepe University, Turkey during the last 3 years. Dent Traumatol.

[ref19] Santos SE, Marchiori EC, Soares AJ, Asprino L, de Souza Filho FJ, de Moraes M (2010). A 9-year retrospective study of dental trauma in Piracicaba and neighboring regions in the State of Sao Paulo, Brazil. J Oral Maxillofac Surg.

[ref20] Segura-Egea JJ, Gould K, Sen BH (2018). European Society of Endodontology position statement: the use of antibiotics in endodontics. Int Endod J.

[ref21] Seppanen L, Rautemaa R, Lindqvist C, Lauhio A (2010). Changing clinical features of odontogenic maxillofacial infections. Clin Oral Investig.

[ref22] Shulman JD, Sauter DT (2012). Treatment of odontogenic pain in a correctional setting. J Correct Health Care.

[ref23] Tulip DE, Palmer NO (2008). A retrospective investigation of the clinical management of patients attending an out of hours dental clinic in Merseyside under the new NHS dental contract. Br Dent J.

[ref24] Tung-Yiu W, Jehn-Shyun H, Ching-Hung C, Hung-An C (2000). Cervical necrotizing fasciitis of odontogenic origin: a report of 11 cases. J Oral Maxillofac Surg.

[ref25] Uluibau IC, Jaunay T, Goss AN (2005). Severe odontogenic infections. Aust Dent J.

[ref26] Veiga NJ, Pereira CM, Ferreira PC, Correia IJ (2014). Oral health behaviors in a sample of Portuguese adolescents: an educational issue. Health Promot Perspect.

[ref27] Warren M, Widmer R, Arora M, Hibbert S (2014). After hours presentation of traumatic dental injuries to a major paediatric teaching hospital. Aust Dent J.

[ref28] Williams DR (2003). The health of men: structured inequalities and opportunities. Am J Public Health.

[ref29] Zaleckiene V, Peciuliene V, Brukiene V, Drukteinis S (2014). Traumatic dental injuries: etiology, prevalence and possible outcomes. Stomatologija.

